# Optimizing the transfusion strategy in surgical patients in a Lebanese university hospital

**DOI:** 10.1186/s13741-024-00374-y

**Published:** 2024-03-15

**Authors:** Stephanie El Hawat, Rita Saliby, Ghassan Sleilaty, Alain El Asmar, Anthony Ghosn

**Affiliations:** 1 School of Medicine and Medical Sciences, Holy Spirit University of Kaslik, P.O. Box 446, Jounieh, Lebanon; 2 Department of Laboratory Medicine, Notre Dame Des Secours University Hospital Center (CHUNDS), P.O. Box 3, Byblos, Lebanon; 3 Beirut, Lebanon; 4 Division of Emergency Medicine, Department of Internal Medicine, School of Medicine, Lebanese American University, Beirut, Lebanon; 5 Department of Anesthesiology, Notre Dame Des Secours University Hospital Center (CHUNDS), P.O. Box 3, Byblos, Lebanon

**Keywords:** Transfusion, Anemia, Iron tests, Transfusion-saving strategy, Surgical bleeding

## Abstract

**Background and purpose:**

Our aim was to analyze factors that influence transfusion requirements in surgical patients in order to achieve a transfusion-saving strategy.

**Methods:**

Data was collected from patient’s files at the Notre Dame de Secours-Jbeil University Hospital Center between January 2017 and June 2019. Selection was made for 400 patients who had undergone surgery and required transfusion. The studied variables were age, sex, and type of surgery whether planned or urgent with its expected level of bleeding. The presence of chronic anemia, coronary artery disease, values of hemoglobin and hematocrit before and after transfusion, iron status preoperatively, and post-operation complications were also noted.

**Results:**

The analysis of 400 transfused surgical patients showed that the mean age was 62 ± 18 years with 52.5% women and 47.5% men. In 82.3% of patients, surgical bleeding was expected, 77.8% of surgeries were scheduled, and 22.3% were urgent. Fifty-two percent of patients were known to have coronary artery disease. Orthopedic (35%) and cardiothoracic (29.5%) surgeries had the highest transfusion rate.

Among all patients, only 106 patients (26.5%) underwent a preoperative iron workup. The pre-transfusion levels of hemoglobin were 9.9 ± 0.6 and hematocrit of 29.7 ± 1.9. 26.3% of patients had a post-transfusion complication.

On the other hand, 19.5% of women and 20% of men were already anemic when admitted to the hospital.

Anemic women required 7.6 times more transfusions than non-anemic, while anemic men required 12.38 times more transfusions than non-anemic men. Age, presence of coronary artery disease, and chronic anemia have been found to be factors increasing the risk of post-transfusion complications. Finally, urgent and unplanned surgeries are 2.9 times more likely to cause post-transfusion complications.

**Conclusion:**

This study therefore confirms that anemic patients are more likely to receive perioperative blood transfusions. Consequently, in order to reduce blood transfusion and its complications, it would be beneficial primarily to diagnose and treat anemia preoperatively. Other transfusion-saving strategies could also be useful in the setting of surgical bleeding, such as the use of tranexamic acid and different autologous transfusion methods like the cell saver.

## Background

Blood transfusion, a key therapeutic element of vital importance intraoperatively and postoperatively, is often necessary and unavoidable, given the hemorrhagic risk associated with certain surgical procedures and allowing the maintenance of hemodynamic stability.

According to the ABC study “Anemia and Blood Transfusion in Critical Care” (Vincent et al. 2003), 42% and 57% of patients who underwent elective surgery and urgent surgery, respectively, received intraoperative or postoperative transfusions.

However, like any procedure, transfusion is not risk-free and it can be associated with many complications ranging from mild to severe sometimes vital. These complications include allergic, febrile, hemolytic, hemodynamic, infectious, and immunological reactions. Unfortunately, blood components are very expensive and supplies are limited these days.

The evaluation of the benefit-risk ratio of the transfusion and the assessment of the clinical status of the patient are of high importance. However, the predictive factors of transfusion remain poorly evaluated in the hospital setting.

On the other hand, anemia is a major preoperative risk factor increasing the need for transfusion. This condition is common regardless of the type of surgery. According to the WHO classification, it affects more than 54.4% of patients who underwent cardiac surgeries and 39% of patients undergoing non-cardiac surgeries (Hans and Jones 2013). Usually, it is diagnosed by anesthesiologists in the perioperative period and can be associated with unfavorable results and prognosis.

Nevertheless, anemia can be modified and avoided by correcting the preoperative hemoglobin count without resorting to transfusion, hence the importance of diagnosing and treating anemia before surgery. This is one of the foundations of the “Patient Blood Management” concept (Lasocki et al. 2016), based on the implementation of transfusion-sparing strategies. Correcting the anemia preoperatively reduces the frequency and the need for transfusion, thus minimizing its side effects.

Many approaches are therefore used to avoid the transfusion of allogeneic blood. They mainly include erythropoietin and iron supplementation, the use of tranexamic acid, and autologous transfusions such as the intraoperative cell-saver machine.

Iron deficiency is one of the most common causes of anemia. Preoperative oral iron supplementation is beneficial in mild to moderate anemia if the time interval before surgery is sufficient (6 to 8 weeks). To note that incremental increases in Hb are slow and small (< 1 g/dL).

In surgical patients, intravenous iron is indicated to correct iron deficiency in the case of:

- Moderate to severe iron deficiency anemia
- Concomitant use of erythropoiesis-stimulating agents
- Ineffectiveness of oral iron or non-tolerance
- Insufficient time before surgery to correct anemia.

In addition, recombinant human erythropoietin (rHuEPO) has been approved by the Food and Drug Administration for the treatment of anemic patients undergoing elective, non-cardiac, and non-vascular surgery and patients at high risk of significant perioperative blood loss, by reducing allogeneic transfusion requirements. Its main disadvantage is its high cost.

Furthermore, the importance of tranexamic acid (TXA) in reducing intraoperative blood loss and lowering transfusion requirements has only recently been recognized. In addition to being inexpensive and having few side effects, it has been shown to be effective and safe as a powerful means of perioperative blood sparing. Another option could be desmopressin (DDAVP), a synthetic vasopressin analog, which is commonly used to treat hemophilia and von Willebrand disease because it increases platelet adhesion and aggregation and stimulates the production of von Willebrand factor. Some professionals advise perioperative DDAVP for patients with bleeding and platelet dysfunction to avoid blood loss and blood product transfusion during surgery. Furthermore, because of its capacity to connect with calcium ions in the blood, vitamin K aids in the initiation of blood clotting components. Before surgery, a doctor could advise the patient to take vitamin K to aid with blood clotting and lessen the chance of excessive bleeding during the procedure.

Nowadays, allogeneic blood has become a very rare and expensive resource. The process involves donor screening and selection and deferring procedures. Screening for transfusion transmissible infections is also a very expensive procedure along with blood typing and cross-matching, blood components processing, storage and distribution, and hospital costs for storing, handling, and transfusing the blood.

For the preoperative autologous donation, the patient presents multiple times before the elective surgery to the laboratory, for repeated blood donations of ∼450 ml. This process begins up to 5 weeks before surgery allowing the collection of two up to four units of blood and is called the “leapfrog” method To maintain erythropoiesis, iron supplementation may be necessary. The last donation must take place at least 48 to 72 h before the surgery to allow rebalancing of the blood volume. Acute normovolemic hemodilution is another technique that can be performed in the operating room, moments after induction of anesthesia. One liter to one and a half liters of blood is collected, and the intravascular volume is concomitantly replaced with either a crystalloid or a colloid, or both, in order to maintain blood volume. The blood collected is carefully labeled and stored without the need for refrigeration, with the patient in the operating room. Towards the end of the surgery and once hemostasis has been achieved, blood can then be transfused into the patient. The blood-saving benefit of hemodilution is the result of reducing the mass of red blood cells lost during surgical bleeding.

Among the strategies mentioned above, the cell saver is the most important and commonly used technique. It is a safe, economical, and relatively inexpensive method of avoiding allogeneic transfusion of red blood cells. This technique can be used during the peri- or postoperative periods allowing the recovery of the patient’s blood during the surgical intervention, and transfusing it after filtration and washing, so keeping only the Red cells.

## Methods

### Study design and participants

A retrospective descriptive study was carried out at the Notre Dame de Secours-Jbeil University Hospital between January 2017 and June 2019 in order to analyze the factors influencing transfusion requirements in surgical patients. Records of 400 patients operated on and transfused between January 2017 and June 2019 at the Notre Dame de Secours-Jbeil University Hospital Center were analyzed.

- ➢ Inclusion criteria:
  - Patients who have undergone any type of surgical intervention and have required a perioperative transfusion.
- ➢ Exclusion criteria:
  - Transfused patients apart from any surgical intervention
  - Pediatric population

The variables studied in our study are age, gender, expected bleeding nature of surgeries, degree of urgency, type of surgery, presence of chronic anemia, presence of known coronary pathology, hematocrit and hemoglobin values upon admission to the hospital, then the values before and after the transfusion, the evaluation of the preoperative iron status, the complications and coagulopathies that occurred post-transfusion, and the hemodynamic instability preoperatively.

Data was analyzed with a confidence interval of 95% and a margin of error of 5%. Quantitative variables are expressed as a percentage; on the other hand, the qualitative variables are expressed as mean ± standard deviation (standard deviation). *P* value < 0.05 was considered statistically significant.

Mann–Whitney *U*-test, chi-square test, and median test were used to calculate descriptive statistics to analyze baseline characteristics and clinical parameters of study participants.

The association between dependent and independent variables was analyzed using a one-way analysis of variance (ANOVA).

## Results

The analysis of the files of 400 operated and transfused patients showed that the average age was 62 ± 18 years with 210 (52.5%) women and 190 (47.5%) men (Table 1). In 329 transfused cases (82.3%), surgery was planned hemorrhagic against 71 cases of unplanned hemorrhagic surgeries (17.7%).

**Table 1:** Main Results by type of patient, type of surgery and pre-op labs

**Sex of patients**	52.5% women	47.5% men
**Type of patients**	52% with coronary artery disease	48% without coronary artery disease
**Type of surgery**	82.3% planned hemorrhagic	17.7% not planned hemorrhagic
**Type of surgery**	77.8% planned	22.3% urgent
**Surgery with the highest transfusion rate**	Orthopedic (35%)	Cardiothoracic (29.5%)
**Preoperative iron level tested**	26.5% YES	73.5 NO
**Pre-op labs**	Hemoglobin of 9.9 ± 0.6	Hematocrit of 29.7 ± 1.9

In 311 cases (77.8%), the surgery was planned against 89 patients (22.3%) of urgent surgeries. Among the 400 operated and transfused patients, 208 (52%) are known to have some coronary artery disease versus 192 patients (48%) with no known coronary problem. Orthopedic (35%) and cardiothoracic (29.5%) surgeries required the most transfusions.

Among all the transfused patients, only 106 patients (26.5%) had their preoperative iron level tested against 294 patients (73.5%) with no iron test done. The assessment before the transfusion showed a hemoglobin of 9.9 ± 0.6 and a hematocrit of 29.7 ± 1.9. In 26.3% of the cases (105 patients), post-transfusion complications were noted, and in 27.8% of the cases (111 patients), a coagulation disorder appeared after the transfusion.

On the other hand, among the 210 transfused women, it was discovered that 41 patients (19.5%) were already anemic on admission, while 38 patients of the 190 men (20%) were anemic upon arrival.

Among the non-anemic women, 26.8% were transfused against 73.2% without any need for transfusion. Whereas in women known to be anemic, the need for transfusion was 73.5% versus 26.5% without transfusion, with a largely significant difference (*p* < 0.0001). Therefore, anemic women needed 7.6 times more transfusions than non-anemics.

Concerning the non-anemic men, 18.2% needed transfusion against 81.8% who did not, while the anemic men needed 73.3% of transfusion versus 26.7% without transfusion need. The difference between the 2 groups was also very significant with *p* < 0.0001. On the other hand, anemic patients required 12.38 times more transfusion than non-anemics.

We also found that age increases the risk of complications and every year increases the risk by 2.5% with a *p* = 0.031. On the other hand, coronary patients presented 4.8% more complications than non-coronary patients with *p* = 0.047. Similarly, patients with chronic anemia presented 30% more transfusion complications with *p* = 0.047. Finally, urgent and unplanned surgeries are 2.9 times more at risk of post-transfusion problems with *p* = 0.016 (Table 2).

**Table 2:** Statistically significant results

	**Number of patients**		
Distribution preoperative anemia—transfusion requirement	79	Independent-samples Mann–Whitney *U* test	< 0.0001
Distribution age—transfusion complications	53	Independent-samples Mann–Whitney *U* test	0.031
Distribution coronary heart disease—transfusion complications	39	Independent-samples Mann–Whitney *U* test	0.047
Distribution chronic anemia—transfusion complications	36	Independent-samples Mann–Whitney *U* test	0.047
Distribution urgent surgeries—transfusion complications	40	Independent-samples Mann–Whitney *U* test	0.016

## Discussion

Our study therefore confirms that anemia is the main predictive factor for transfusion, thus increasing the risk of various post-transfusion complications during the perioperative phase. In order to eliminate any factor that could interfere with our results, among our exclusion criteria is the elimination of patients who received transfusions without any surgical intervention and the pediatric population, as the indications and transfusion thresholds differ from those in adults.

According to our study, 19.5% of women and 20% of men have preoperative anemia. Indeed, according to Rosenthal et al. (2019), 14 to 40% of patients are anemic before their interventions. In addition, Hong et al. (2017) conducted a study between July 2013 and June 2014 to establish the prevalence and cause of preoperative anemia in patients scheduled for major surgery. Of the 1494 patients, 13.9% had preoperative anemia, with a male predominance (70.7%), and 27.4% of them suffered from iron deficiency anemia.

Our results showed that anemia exposes to a greater risk of receiving blood transfusions, converged with the literature.

Fowler et al. (2015) studied the association between preoperative anemia and its postoperative consequences. A meta-analysis of 24 studies analyzing more than 949,445 patients was carried out for this purpose. According to the WHO definition, 21,322 (29.9%) of 71,338 patients were anemic. The incidence of transfusion was much higher in anemic patients 15,804 (18.5%) versus 9539 (4.7%) in non-anemic patients. This is consistent with the results of our study where anemic patients required an average of 8.68 times more transfusion than non-anemics. According to this study, anemia and transfusions were associated with increased mortality (*P* < 0.001) and morbidity after surgery. However, Fowler et al. did not investigate whether correction of preoperative anemia might improve outcomes.

Anemia also caused, according to Musallam et al. (2011), a synergistic negative effect greater than tenfold on the results, when present with any other known preoperative risk factor.

Based on the same principle, Klein et al. (2016) studied the prevalence and importance of anemia in cardiac surgery in 19,033 patients operated on in 12 centers between 2010 and 2012 in the UK. This study showed that 31% of patients had preoperative anemia and were therefore more likely to receive a transfusion. These patients also had longer lengths of stay in intensive care units and hospitals, with a doubled risk of death after heart surgery. Therefore, according to Klein et al., the diagnosis and treatment of preoperative anemia is an important area of research with the potential to reduce mortality.

According to our study, a liberal transfusion strategy was adopted at the hospital with a transfusion threshold of 9.9 ± 0.6. In 2012, Carson et al. (2012) identified nineteen randomized studies involving a total of 6264 patients comparing a restrictive or liberal transfusion strategy. The transfusion threshold in the restrictive strategies varied between 70 and 90 g/l. They found that restrictive transfusion strategies reduced the need for red blood cell transfusion by 39%. A weak point to mention is the heterogeneity concerning the threshold used for the liberal strategy by the different studies and the absence of data concerning patients with coronary syndrome.

However, Docherty et al. (2017) suggest that there is no difference in 30-day mortality between restrictive and liberal transfusion thresholds, after a systematic review of 11 randomized studies including patients with cardiovascular disease. The restrictive transfusion threshold for most included trials was 8 g/dl compared to a liberal transfusion threshold of 10 g/dl. In contrast, they noted an increased risk of the acute coronary syndrome in cardiac patients who were randomized to a restrictive blood transfusion threshold. They therefore conclude that it would be plausible that patients with cardiovascular disease could benefit from higher transfusion thresholds than non-cardiac patients. Finally, they propose that a more liberal transfusion threshold (> 80 g/l) in this population be used until a high-quality study including endpoints for longer-term mortality, acute coronary syndrome, quality of life, and cost-effectiveness has been achieved.

Our study also showed that age increases the risk of post-transfusion complications. On the other hand, for De Santo et al. (2017), the complications and the post-transfusion mortality rate were significantly higher in all the groups transfused following cardiac surgery, independently of age, but elderly patients had a 1.3-fold increase in the relative risk of transfusion (*P* < 0.001).

Supporting our correlation of urgent surgeries with increased complications following transfusion, Havens et al. (2016) conducted a study on 992 patients, 33% of whom underwent unscheduled surgery. Despite similar blood loss, emergency-operated patients received higher rates of intraoperative blood product transfusion, which was therefore independently associated with major postoperative complications.

By comparing the risk of complications, our study showed that coronary patients had more post-transfusion problems. In this same context, Rao et al. (2004) found that blood transfusion in the context of coronary syndromes is associated with a higher rate of mortality and myocardial infarction.

Consequently, in view of all the harmful effects that flow from transfusion, its use as a treatment for anemia capable of improving the general condition of the patient and speeding up his recovery has been replaced by a more rational view according to which other less risky methods should be used. Additionally, increased demand in an aging population and declining numbers of blood donors have made it even more imperative to take a different approach to transfusion. Hence, the crucial importance of PBM “Patient Blood management”.

Supporting this principle, Faulds et al. (2019) conducted a retrospective study of 15,245 patients to assess the value of PBM in detecting and managing preoperative anemia before elective surgery. They found that the transfusion rate was 9.2% in anemic patients assessed by PBM significantly lower than that of non-PBM patients which was 17.4%.

Therefore, the presence of preoperative anemia should be assessed as soon as possible by performing an iron test level before any planned hemorrhagic surgery. And as found by Goodnough et al. (1992), one-third of anemic patients scheduled for elective surgery had iron deficiency anemia. And in our study, 26.5% of patients were investigated by an iron balance requested by their surgeon, aware of the significant impact of the diagnosis of preoperative anemia on the surgeries planned to be hemorrhagic, while hoping to work on increasing this figure in order to detect any candidate PBM patient before planned surgery who is hemorrhagic.

In contrast, Jin et al. (2019) implemented a protocol to facilitate preoperative identification and treatment of anemia by performing iron balance in 87% of anemic patients before their surgeries. Seventy-nine percent of patients with iron deficiency anemia received iron intravenously with ferric carboxymaltose or iron sucrose. They noticed that most patients (72%) had an increase in hemoglobin, averaging 0.98 g/dl. Moreover, they note that this improvement progressed linearly with time, so that a longer time interval allowed a greater increase in hemoglobin.

In this study, PBM was then associated with lower rates of anemia on the day of surgery, transfusion, and volume transfused per patient. For example, in hip surgery, the percentage of patients with immediate preoperative anemia decreased from 17.6 to 12.9% and the transfusion rate was significantly reduced from 21.8 to 15.7%.

The weak point of our study lies in the fact that we were unable to study the cell saver technique used in our hospital, especially in cardiac surgery, due to an insufficient number of patients and therefore which will have no value statistically and clinically significant.

## Conclusion

This study therefore confirms that anemic patients are at higher risk of receiving intraoperative blood transfusions. Therefore, to reduce transfusion requirements and all associated complications, it would then be beneficial to first diagnose and treat anemia preoperatively. Since iron deficiency is one of the main causes, an iron test should be carried out before any scheduled surgery in anemic patients. Treatment modalities mainly include the use of intravenous iron and erythropoietin.

## Data Availability

The data that support the findings of this study are available from the archive of the hospital Notre dame des secours but restrictions apply to the availability of these data, which were used under license for the current study, and so are not publicly available. Data are however available from the authors upon reasonable request and with permission of the medical director.
